# Ontario Veterinary College Society

**Published:** 1895-01

**Authors:** 


Secretary.

### ONTARIO VETERINARY COLLEGE, TORONTO, CANADA.

THE Veterinary Medical Society connected with the above college has held semi-weekly meetings during the session, which have been very interesting and profitable to the members. Among the many good subjects brought forward which may be mentioned were the following essays: "Coal Tar and Its Products," Mr. W. L. West; "Diseases of Joints," Mr. F. V. Barrett; "Tuberculin as a Diagnostic Agent," Mr. H. D. Stebbins; "Physiology of the Kidney," Mr. H. Busman; "Eserine," Mr. L. J. Allen; "Origin of the Horse," Mr. R. C. Hill; "Distemper of the Dog," Mr. G. W. Harrison; "Tetanus," Mr. Wm. Fotheringham; "Cæsarean Section," Mr. W. R. L. Best; "Texas Fever," Mr. E. M. Hendy; "Parturient Eclampsia," Mr. H. S. Creighton; "Rot in Sheep," Mr. D. M. Waldo; "Lameness," Mr. J. M. Farquhar; "Simple and Periodic Ophthalmia," Mr. F. G. Shepard; "Foot Rot in Sheep," Mr. A. A. Moody; "Glanders," Mr. C. L. Megowan; "Cerebro-spinal Meningitis," Mr. A. J. Gibbons. The discussion which the different subjects brought forth were also interesting and instructive, and by their character indicated the general knowledge of the various subjects of the members, which augurs well for their success as practitioners.

The office-bearers for the session are: *President*—Prof. Andrew Smith, F.R.C.V.S.; *Secretary*—Edw. T. Handy; *Assistant Secretary*—H. E. Hensel; *Treasurer*—G. J. Brossar; *Librarian*—H. W. Witmer.

